# Supplementary material for: Droplet-based single-cell joint profiling of histone modifications and transcriptomes
Source: Nat Struct Mol Biol. 2023 Aug 10;30(10):1428–33. doi: 10.1038/s41594-023-01060-1 (PMC10584685; doi:10.1038/s41594-023-01060-1)
Supplement: Supplementary file 1 — Reporting Summary [file 41594_2023_1060_MOESM1_ESM.pdf]

nature portfolio

Corresponding author(s): Bing Ren

Last updated by author(s): Jul 1, 2023

Reporting Summary

Nature Portfolio wishes to improve the reproducibility of the work that we publish. This form provides structure for consistency and transparency in reporting. For further information on Nature Portfolio policies, see our Editorial Policies and the Editorial Policy Checklist.

Statistics

For all statistical analyses, confirm that the following items are present in the figure legend, table legend, main text, or Methods section.

n/a	Confirmed	
☐	☒	The exact sample size (*n*) for each experimental group/condition, given as a discrete number and unit of measurement
☐	☒	A statement on whether measurements were taken from distinct samples or whether the same sample was measured repeatedly
☐	☒	The statistical test(s) used AND whether they are one- or two-sided<br>*Only common tests should be described solely by name; describe more complex techniques in the Methods section.*
☒	☐	A description of all covariates tested
☐	☒	A description of any assumptions or corrections, such as tests of normality and adjustment for multiple comparisons
☐	☒	A full description of the statistical parameters including central tendency (e.g. means) or other basic estimates (e.g. regression coefficient) AND variation (e.g. standard deviation) or associated estimates of uncertainty (e.g. confidence intervals)
☐	☒	For null hypothesis testing, the test statistic (e.g. *F*, *t*, *r*) with confidence intervals, effect sizes, degrees of freedom and *P* value noted<br>*Give P values as exact values whenever suitable.*
☒	☐	For Bayesian analysis, information on the choice of priors and Markov chain Monte Carlo settings
☒	☐	For hierarchical and complex designs, identification of the appropriate level for tests and full reporting of outcomes
☐	☒	Estimates of effect sizes (e.g. Cohen's *d*, Pearson's *r*), indicating how they were calculated

Our web collection on statistics for biologists contains articles on many of the points above.

Software and code

Policy information about availability of computer code

Data collection	Illumina bcl2fastq2
Data analysis	10XGenomics cellranger (v2.0.0), 10XGenomics cellranger-atac (v2.0.0), 10XGenomics cellranger-arc (v6.1.2), HOMER (v4.11), MACS2 (v2.1.2), Samtools (v1.14), Seurat (v4.1.0), Signac (v1.6.0), deepTools (v3.5.1), IGV (v.2.15.4), Picard (v2.25.0), Monocle3 (v1.2.7), pairtools (v1.0.2), bedtools (v2.27.1), Juicer tools (v1.22.01), PANTHER (v17.0)<br>Custom scripts and code to reproduce figures are available at: https://github.com/Xieeeee/Droplet-Paired-Tag

For manuscripts utilizing custom algorithms or software that are central to the research but not yet described in published literature, software must be made available to editors and reviewers. We strongly encourage code deposition in a community repository (e.g. GitHub). See the Nature Portfolio guidelines for submitting code & software for further information.

Data

Policy information about availability of data

All manuscripts must include a data availability statement. This statement should provide the following information, where applicable:

- Accession codes, unique identifiers, or web links for publicly available datasets
- A description of any restrictions on data availability
- For clinical datasets or third party data, please ensure that the statement adheres to our policy

Raw data obtained in this study have been deposited at the NCBI Gene Expression Omnibus (GEO) (http://www.ncbi.nlm.nih.gov/geo/) with accession number

GSE224560. The processed data can also be accessed as supplementary files in GEO. Datasets for mESC H3K27ac ChIP-seq were downloaded from 4DN data portal with the accession numbers 4DNESTVGLCD9. Other external datasets were downloaded from NCBI Gene Expression Omnibus (GEO) with the following accession numbers: mESC H3K27me3 ChIP-seq (GSE156589), Paired-Tag (GSE152020), CoTECH (GSE158435), scCUT&Tag on PMBC (GSE157910), scCUT&Tag on brain (GSE163532), scCUT&Tag-pro (GSE195725), snm3C-seq on brain (GSE156683) and ChIP-seq on mouse cortex excitatory neurons (GSE141587). The CEMBA snATAC-seq datasets and BICCN 10XsnRNA-seq MOp data are downloaded via the NeMO archive (RRID: SCR_016152) (https://assets.nemoarchive.org/dat-ch1nqb7). 10x PBMC scRNA-seq and E18 embryonic mouse brain Multiome datasets were downloaded from the 10x Genomics website (https://www.10xgenomics.com/resources/datasets). Source data for statistical analysis are provided along in this paper.

Research involving human participants, their data, or biological material

Policy information about studies with human participants or human data. See also policy information about sex, gender (identity/presentation), and sexual orientation and race, ethnicity and racism.

Reporting on sex and gender	N/A
Reporting on race, ethnicity, or other socially relevant groupings	N/A
Population characteristics	N/A
Recruitment	N/A
Ethics oversight	N/A

Note that full information on the approval of the study protocol must also be provided in the manuscript.

Field-specific reporting

Please select the one below that is the best fit for your research. If you are not sure, read the appropriate sections before making your selection.

☒ Life sciences ☐ Behavioural & social sciences ☐ Ecological, evolutionary & environmental sciences

For a reference copy of the document with all sections, see nature.com/documents/nr-reporting-summary-flat.pdf

Life sciences study design

All studies must disclose on these points even when the disclosure is negative.

Sample size	Sample size was determined based on prior published data from similar experiments (Preissl et.al., Nat. Neuroscience, 2018; Cao et.al., Science, 2018). To evaluate robustness of the methods, each set of experiment was carried out with tissues samples dissected from three individuals.
Data exclusions	Low quality single nuclei (low number of reads / FRiP, low number of genes captured) were excluded from downstream analysis as outlined in the Methods section. Genes (GENCODE vm25) with sufficient levels of transcription (RPKM > 1), or genomic regions with sufficient levels of epigenomic signal (RPKM > 1 for already identified cCREs) were retained for subsequent analysis.
Replication	Three biological replicates were performed for each set of experiment. All datasets from independent replicates showed similar results.
Randomization	Allocation was random.
Blinding	The experiments were not blinded since identities of histone modification targets and tissue regions are needed to evaluate the specificity and sensitivity of the method. Clustering of single-nuclei transcriptome and epigenome data were unsupervised.

Reporting for specific materials, systems and methods

We require information from authors about some types of materials, experimental systems and methods used in many studies. Here, indicate whether each material, system or method listed is relevant to your study. If you are not sure if a list item applies to your research, read the appropriate section before selecting a response.

Materials & experimental systems

n/a	Involved in the study
☐	☒ Antibodies
☐	☒ Eukaryotic cell lines
☒	☐ Palaeontology and archaeology
☐	☒ Animals and other organisms
☒	☐ Clinical data
☒	☐ Dual use research of concern
☒	☐ Plants

Methods

n/a	Involved in the study
☒	☐ ChIP-seq
☐	☒ Flow cytometry
☒	☐ MRI-based neuroimaging

Antibodies

Antibodies used	Antibodies used in this study include: H3K27ac (Abcam, ab177178, Lot GR3202987-20 (recombinant); Abcam, ab4729, Lot GR3442886-1 (polyclonal)) and H3K27me3 (Abcam, ab192985, Lot GR3399022-3 (recombinant)). For all assays, 1 µg of antibody was used for 200 - 500k nuclei, in 75 µL reaction system.
Validation	All antibodies used in this study are commercially available, and have been validated appropriate for the assays used in this manuscript by the provider (https://www.abcam.com/products/primary-antibodies/histone-h3-acetyl-k27-antibody-ep16602-chip-grade-ab177178.html; https://www.abcam.com/products/primary-antibodies/histone-h3-acetyl-k27-antibody-chip-grade-ab4729.html; https://www.abcam.com/products/primary-antibodies/histone-h3-tri-methyl-k27-antibody-epr18607-chip-grade-ab192985.html).<br>We also validated all antibodies with in-house CUT&Tag and compared to ENCODE data(H3K27ac (ENCSR000AOC), H3K27me3 (ENCSR000DTY)) before using for publication.<br>We found that antibodies specificity is critical for high-quality signals of single-cell histone data. For H3K27ac, although recombinant antibody yielded a higher fragment number per cell than polyclonal antibodies, its enrichment at transcription starting sites or ChIP-seq peaks was lower. Therefore, except for replicate 1 (rep1) of the mouse frontal cortex datasets, all other experiments targeting in H3K27ac were carried out with the polyclonal antibody.

Eukaryotic cell lines

Policy information about cell lines and Sex and Gender in Research

Cell line source(s)	Mouse embryonic stem cells (mESC) used in this study is genetically engineered to have allelic tagging of Sox2 genes with egfp and mcherry on CAST and 129/Sv allele, respectively, and also harbors an insertion of four CTCF binding sites in between Sox2 and its downstream super-enhancer on the CAST allele (Huang et.al., 2021, Nat. Genetics). The parental, hybrid F123 mESC line (F1 Mus musculus castaneus×S129/SvJae, maternal 129/Sv, paternal CAST) was from Rudolf Jaenisch's laboratory at the Whitehead Institute at MIT.
Authentication	Cells were not authenticated.
Mycoplasma contamination	Cells were not tested for mycoplasma.
Commonly misidentified lines<br>(See ICLAC register)	None of the cell lines used are listed in the ICLAC database.

Animals and other research organisms

Policy information about studies involving animals; ARRIVE guidelines recommended for reporting animal research, and Sex and Gender in Research

Laboratory animals	All animal work described in this manuscript has been approved and conducted under the oversight of the UC San Diego Institutional Animal Care and Use Committee. Male C57BL/6J mice were purchased from the Jackson Laboratory (#000664) at 12 weeks of age and housed in the barrier facility at UC San Diego in a 12-hour light/dark cycle in a temperature-controlled room, with ad libitum access to water and food, until euthanasia and tissue collection at 16 weeks of age. The temperature in the animal facility is maintained within the range of 68°F to 72°F, while the humidity levels can vary between 35% and 60%. The frontal cortex was dissected from 16-week male mice, snap-frozen in liquid nitrogen, and stored at -80°C before proceeding to nuclei extraction.
Wild animals	The study did not involve wild animals.
Reporting on sex	All three replicates used in this study are male C57BL/6J mice
Field-collected samples	The study did not involve field collected samples
Ethics oversight	All animal work described in this manuscript has been approved and conducted under the oversight of the UC San Diego Institutional Animal Care and Use Committee.

Note that full information on the approval of the study protocol must also be provided in the manuscript.

Flow Cytometry

Plots

Confirm that:

☒ The axis labels state the marker and fluorochrome used (e.g. CD4-FITC).

☒ The axis scales are clearly visible. Include numbers along axes only for bottom left plot of group (a 'group' is an analysis of identical markers).

☒ All plots are contour plots with outliers or pseudocolor plots.

☒ A numerical value for number of cells or percentage (with statistics) is provided.

Methodology

Sample preparation	Single cell suspensions were prepared from frozen mouse cortex by douncing in the douncing buffer (0.25 M sucrose (Sigma, S7903), 25 mM KCl (Sigma, P9333), 5 mM MgCl2, 10 mM Tris-HCl pH 7.4, 1 mM DTT (Sigma, D9779), 1× Protease Inhibitor, 0.5 U µl−1 Rnase OUT, 0.5 U µl−1 SUPERase Inhibitor). The cell suspension was then filtered through a 30-µm Cell-Tric (Sysmex) for debris removal, and spun down for 10 min at 300g, 4 °C. Cell pellets were washed once with douncing buffer, spun down again, and resuspended in cold nuclei permeabilization buffer for 10mins. Permeabilized nuclei were pelleted by centrifuge for 10 min at 1,000g, 4 °C, and washed with sort buffer (1× PBS (Gibco, 10010023), 1×Protease Inhibitor (Roche, 05056489001), 0.5 U µl−1 Rnase OUT (Invitrogen, 10777-019), 0.5 U µl−1 SUPERase Inhibitor (Invitrogen, AM2694), 1mM EDTA (Invitrogen, 15575020), 1% BSA (Sigma, A1595)) once. After resuspension in sort buffer, nuclei are stained with 2 µM 7-AAD (Invitrogen, A1310) for 10mins on ice, and proceed to Fluorescence-activated cell sorting (FACS) with an SH800 cell sorter (Sony) for isolation of single nucleus.
Instrument	SH800 Cell Sorter (Sony)
Software	Data analysis and display are performed in the SH800 software.
Cell population abundance	We used 7-AAD to stain intact nuclei and separated them from debris, without separating identities of different cell types. Using 'normal' mode in sorting nuclei, we collected 200,000-500,000 nuclei for each sample for down-streaming processing.
Gating strategy	First, potential nuclei were identified using forward scatter (FSC) area and back scatter (BSC) area. Next, potential doublets were removed based on BSC and FSC signal width. Finally, diploid nuclei (2n) were sorted into each tube for down-streaming processing.

☒ Tick this box to confirm that a figure exemplifying the gating strategy is provided in the Supplementary Information.
