# Supplementary material for: Droplet-based single-cell joint profiling of histone modifications and transcriptomes
Source: Nat Struct Mol Biol. 2023 Aug 10;30(10):1428–33. doi: 10.1038/s41594-023-01060-1 (PMC10584685; doi:10.1038/s41594-023-01060-1)
Supplement: Supplementary file 4 — Step-by-step protocol of Droplet Paired-Tag. [file 41594_2023_1060_MOESM4_ESM.docx]

**Droplet-based Single-cell Joint Profiling of Histone Modification and Transcriptome**

Yang Xie^1,2,7^, Chenxu Zhu^3,4,5,7^, Zhaoning Wang^1^, Melodi Tastemel^1^, Lei Chang^1,3^, Yang Eric Li^1,3^, Bing Ren^1,3,6,*^

^1^Department of Cellular and Molecular Medicine, University of California, San Diego, San Diego, CA, USA

^2^Biomedical Sciences Graduate Program, University of California, San Diego, La Jolla, CA, USA

^3^Ludwig Institute for Cancer Research, La Jolla, CA, USA

^4^New York Genome Center, New York, NY, USA

^5^Department of Physiology and Biophysics, Institute for Computational Biomedicine, Weill Cornell Medicine, New York, NY, USA

^6^Center for Epigenomics, Institute of Genomic Medicine, Moores Cancer Center, University of California San Diego, School of Medicine, La Jolla, California, USA

^7^These authors contributed equally

*Correspondence: [biren@health.ucsd.edu](mailto:biren@health.ucsd.edu)

**Abstract**

We introduce Droplet Paired-Tag, a multiomic technique which enables rapid and convenient mapping of histone modifications and gene expression simultaneously at single-cell resolution on a large scale. Compared to other techniques, Droplet Paired-Tag shows outstanding efficacy in reconstructing cell type-specific active or repressive gene regulatory networks in both cultured mammalian cells and primary mouse brain tissues.

**Introduction**

The regulation of gene expression patterns in multicellular eukaryotic organisms is governed by chemical modifications to histone proteins and nucleic acids, collectively known as the epigenome^1^. Analyzing the epigenome has been proven valuable in revealing the underlying mechanisms of gene regulation and its dysregulation in development, disease, and aging. However, profiling the epigenome of complex tissues presents challenges due to the presence of diverse cell types and states within the samples. To overcome these challenges, single-cell multiomic assays have emerged, enabling the simultaneous investigation of multiple molecular modalities^2^. These assays open the possibility to unravel the intricate interplay between the epigenome and the transcriptional machinery at a single-cell level. Nonetheless, the adoption of current single-cell epigenomics assays has been limited due to factors such as time-consuming procedures, complicated manipulation and limited accessibility.

In this study, we introduce Droplet Paired-Tag, a rapid and widely accessible technique for joint profiling of histone modifications and transcriptomes at the single-cell level. We have made significant modifications to the original combinatorial indexing-based Paired-Tag protocol. The key enhancements include nuclei barcoding using a commercially available microfluidics platform (specifically, the 10x Chromium Single Cell Multiome) and simplifying the library preparation process. Droplet Paired-Tag outperforms the conventional Paired-Tag approach by providing a higher signal-to-noise ratio for histone modification data. Moreover, it enables the generation of joint modality data with the same efficiency as single-modality methods, such as scRNA-seq and scCUT&Tag.

**Reagents**

- - PBS (Thermo Fisher Scientific, Cat#10010-23)
  - Tris-HCl (Sigma, Cat#S7653)
  - MgCl_2_ (Sigma, Cat#63069)
  - NaCl (Sigma, Cat#S7653)
  - Protein A-Tn5 (MacroLab, p2409)
  - cOmplete EDTA-free Protease Inhibitor Cocktail (Roche, Cat#05056489001)
  - RNaseOUT (Invitrogen, Cat#10777-019)
  - SUPERaseIN (Invitrogen, Cat#AM2694)
  - IGEPAL CA-630 (Sigma, Cat#I8896)
  - Phosphate Buffered Saline with 10% Bovine Albumin (10%BSA) (Sigma, Cat#SRE0036)
  - Tween-20 (Bio-Rad, Cat#1662404EDU)
  - HEPES (Invitrogen, Cat#15630106)
  - Spermidine (Sigma, Cat#85558)
  - Digitonin (Sigma, Cat#D141)
  - EDTA (Invitrogen, Cat#AM9261)
  - SPRI beads (Beckman coulter, Cat#B23319)
  - Chromium Next GEM Single Cell Multiome ATAC + Gene Expression Reagent Bundle (10X Genomics, Cat#1000283)
  - Single Index Kit N Set A, 96 rxn (10X Genomics, Cat#1000212)
  - Dual Index Kit TT Set A 96 rxns (10X Genomics, Cat#1000215)
  - Chromium Next GEM Chip J Single Cell Kit, 48 rxns (10X Genomics, Cat#1000234)
  - Oligos (see Supplementary Table1) for transposome assembly: pMENTs-Bridge, AdapterA, AdapterB (synthesized from IDT)

***The reagents in this section can be stored at 4 °C for up to 3 months.***

- - 1M Spermidine: 145 mg in 1 mL ultrapure H_2_O.
  - 10% IGEPAL-CA630: 100 µL + 900 µL ultrapure H_2_O.
  - 2% Digitonin: 20 mg in 1 mL DMSO.
  - 250 mM MgCl_2_: 250 µL MgCl_2_ (1 M) + 750 µL ultrapure H_2_O.
  - 2X Stop Solution: 10 µL 1M Tris-HCl + 40 µL 500mM EDTA + 200 µL 10%BSA + 750 µL ultrapure H_2_O.
  - Resuspension buffer (ATAC-RSB buffer): 300 µL 1M Tris-HCl (pH 7.4), 60 µL 5M NaCl, 90 µL 1M MgCl2 and 29.55 **mL** H_2_O.
  - 50X Protease Inhibitor (50X PI): 1 tablet of cOmplete EDTA-free Protease Inhibitor Cocktail + 1 mL ultrapure H_2_O.

***The reagents in this section can be stored at -20 °C for up to 1 month.***

- - Bridged Protein A-Tn5:

1. Mix the oligos in two separated PCR tube: 25 µL AdapterA (100 µM) + 25 µL bridge-pMENTs (100 µM), or 25 µL AdapterB (100 µM) + 25 µL bridge-pMENTs (100 µM).
2. Heat the mixtures for 5 mins in thermal cycler at 95 °C and slowly cool down to 12 °C at the speed of 0.1 °C/s.
3. Mix 1 µL of annealed transposome DNA separately with 6 µL of unloaded Protein A-Tn5 (pA-Tn5, 0.5 mg/mL), pipetting mix and quickly spun down.
4. The mixtures were incubated at room temperature for 30 min then on ice for an additional 10 min. Mix equal volume of assembled pA-Tn5-AdapterA and pA-Tn5-AdapterB, stored at -20 °C.

**Equipments**

- - Benchtop centrifuge (Eppendorf)
  - T100 Thermal Cycler (Bio-Rad)
  - ThermoMixer (Eppendorf)
  - Swing bucket centrifuge (ThermoFisher)
  - Qubit Fluorometric Quantification (ThermoFisher)
  - Magnetic stands (96-well plate stand, PCR-tube stand)
  - Fragment analyzer/Tapestation (Agilent)
  - Micropipetts (0.5-20 μl, 10-200 μl, 100-1000 μl)
  - DNA LoBind tubes (1.5 mL, Eppendorf #L200934G)
  - Maximum recovery tubes (1.5 mL, Axygen, MCT150LC)

**Procedures**

**A. Day 1: nuclei preparation and antibody staining**

A-1 Freshly prepare the following buffer:

1. Prepare nuclei lysis buffer (OMNI buffer) as:

693.75 µL ATAC-RSB buffer

3.75 µL Digitonin (2%)

7.5 µL IGEPAL-CA630 (10%)

7.5 µL Tween-20 (10%)

15 µL SUPERaseIN

7.5 µL IN RNaseOUT

15 µL 50X PI

1. Prepare the high salt Med Buffer #1 (MED1) as:

20 µL HEPES (pH 7.5, 1 M)

54.2 µL NaCl (5 M)

0.5 µL Spermidine (1 M)

20 µL 50X PI

50 µL SUPERaseIN

25 µL RNaseOUT

1 µL IGEPAL-CA630 (10%)

5 µL Digitonin (2%)

4 µL EDTA (500 mM)

200 µL 10% BSA

620.3 µL H_2_O

*(****Note****: RNase inhibitor concentration in this protocol is doubled, which is necessary for sample like mouse embryos. However, reducing the RNase inhibitors concentration to 50% has no impact on the RNA quality for samples like cultured cells or mouse brain.)*

A-2 Antibody + pA-Tn5 conjugation:

1. Mix rabbit polyclonal antibody against specific histone modification epitope (1 µg) with 1µL assembled pA-Tn5 (0.4 mg/ml) in 50 µL MED1 buffer.
2. Rotate 1 hour at room temperature for antibody-pA-Tn5 conjugation.

A-3 Nuclei preparation (for cultured cells):

1. Disassociated the cells according to culture protocol. For each experiment 0.25 - 1 million cells can be used.
2. Spin down cells at 300 RCF for 5 mins, washed with PBS once and spin down again. Resuspend the cells in 750 µL (<5 million cells / 1 mL lysis buffer) OMNI buffer. Sit on ice for 5 min.
3. Spin down nuclei at 1,000 RCF for 10 mins at 4°C and resuspend nuclei in 100 µL MED1 Buffer. Count the nuclei using cell counter.

A-4 Epitope targeting:

1. Aliquote 0.25 - 0.5 million nuclei into Maximum Recovery tubes. Spin down the nuclei at 1,000 RCF for 10 min at 4°C and resuspended in 25 µL MED1 Buffer.
2. Add MED1 buffer with conjugated antibody-pA-Tn5 to each tube, incubate the mixture with rotation at 4 °C overnight.

**B. Day2: multi-omics tagging and nuclei barcoding**

B-1 Freshly prepare the following buffer:

1. Prepare the Med Buffer #2 (MED2) as:

20 µL HEPES (pH 7.5, 1 M)

54.2 µL NaCl (5 M)

0.5 µL spermidine (1 M)

20 µL 50X PI

50 µL SUPERaseIN

25 µL RNaseOUT

1 µL IGEPAL-CA630 (10%)

5 µL digitonin (2%)

200 µL 10% BSA

699.3 µL H_2_O

1. Prepare the PBS + RI for washing as:

20 µL 50X PI

25 µL SUPERaseIN

12.5 µL RNaseOUT

942.5 µL 1X PBS

1. Prepare 1X Nuclei buffer as:

5 µL 20X Nuclei Buffer (10X Genomics PN-2000207)

1 µL DTT (100mM)

5 µL SUPERaseIN

2.5 µL RNaseOUT

86.5 µL H_2_O

B-2 Nuclei washing and tagmentation:

1. Spin down nuclei at 300 RCF for 10 mins at 4°C, washed with 50 µL MED2 buffer. Repeat washing for twice.
2. Resuspend washed nuclei in 50 µL MED2 buffer. Add 2 µL 250 mM MgCl_2_, rotate in ThermoMixer at 550 r.p.m., 37 °C, for 1 hr. During this time, thaw 10X Genomics Single Cell Multiome ATAC + GEX Gel Beads at and barcoding reagents at room temperature.
3. Terminate tagmentation by adding 50 µL 2X Stop Solution, mixing well. Spin down nuclei at 500 RCF for 6 mins at 4°C, wash once with PBS + RI.
4. Spin down nuclei at 500 RCF for 6 mins at 4°C, resuspend in 20 µL 1X Nuclei buffer. Count nuclei using cell counter, aliquot 10 - 16k nuclei into PCR tube, add 1X nuclei buffer to 8 µL.
5. Mix aliquoted nuclei with 7 µL ATAC Buffer B (10X Genomics PN-2000193), directly proceed to droplet generation.

B-3 Droplet generation, reverse transcription and cell barcoding in droplet:

1. Prepare reverse transcription and cell barcoding master mix as:

49.5 µL Barcoding Reagent Mix (10X Genomics PN-2000267)

1.1 µL Template Switch Oligo (10X Genomics PN-3000228)

1.9 µL Reducing Agent B (10X Genomics PN-2000087)

7.5 µL Barcoding Enzyme Mix (10X Genomics PN-2000266)

1. Add 60 µL prepared master mix to 15 µL nuclei mixture, loaded onto Chromium Next GEM Chip J and proceed to droplet generation with ChromiumX microfluidics system (10X Genomics). Detailed processes of chip assembly and reagents adding are the same as described in 10X Multiome user guide (10X Genomics CG000338).
2. After droplet generation completed, aspirate 100 µl emulsions (nuclei-encapsulating droplets) into a new PCR tube. Incubated in a Bio-Rad T100 thermal cycler with following program, with lid temperature set to 50 °C: (Reverse transcription as well as cell barcoding complete during the incubation.)

37 °C 45mins

25 °C 30mins

hold at 4 °C

1. After incubation, add 5 µl Quenching Agent (10X Genomics PN-2000269) to stop the reaction. Slowly pipette mixing well, and proceed to products cleanup.

B-4 Products cleanup:

1. The protocol of products cleanup using Dynabeads and SPRI beads is the same as described in 10X Multiome user guide (10X Genomics CG000338).

**C. Day3: Pre-amplification and DNA / RNA library preparation**

1. The protocol of pre-amplification is almost the same as described in 10X Multiome user guide (10X Genomics CG000338). The modified step is that: during SPRI beads cleanup after pre-amplification, sample is eluted in 80 µL instead of 160 µL. We observed that tagmentated fragments per cell for histone modification CUT&TAG is significantly lower compared to ATAC. Concentrated volume during elution could increase input amount and thus library complexity for DNA library generation.

***Pause Point:*** Pre-amplified products can be stored at -20 °C for up to 6 months.

1. For DNA library preparation, 40 µL purified product is used; For RNA library preparation, 17.5 µL purified product + 17.5 µL H_2_O is used to keep input volume amount the same as 10X Multiome protocol. Detailed protocol of library preparation is the same as described in 10X Multiome user guide (10X Genomics CG000338).

**D: Library sequencing**

1. DNA and RNA libraries are sequenced in separated runs on the Illumina Nextseq2000 instrument. For DNA libraries, 200 cycles kit (Illumina #20046812) is used, with cycle number set as 100+8+24+100 (Read1 100 cycles; Index1 8 cycles; Index2 24 cycles; Read2 100 cycles). Index2 read contains cellular barcodes information. For RNA library, 100 cycles kit (Illumina #20046811) is used, with cycle number set as 28+10+10+72 (Read1 28 cycles; Index1 10 cycles; Index2 10 cycles; Read2 72 cycles). Read1 read contains cellular barcodes information.
2. Sequencing data are demultiplexed using *cellranger-arc mkfastq* (10X Genomics), and then analyzed with in-house pipeline. The analysis scripts and notebooks can be found at: <https://github.com/Xieeeee/Droplet-Paired-Tag>.

**Troubleshooting**

- - Typically, 30-60% of input nuclei can be harvested right before loading the microfluidics system. If only very few nuclei left, (1). considering increasing the number of input nuclei to > 250k; (2). check whether nuclei clumping is observed during the CUT&Tag process. Fragile nuclei may be easier to lyse and aggregate into clump in the high salt buffer, considering crosslink nuclei with 0.1% formaldehyde 5mins at room temperature.
  - Compared to snATAC-seq library, complexity of histone modification library is lower and thus require increased number of indexing PCR cycles. For instance, when loading 15k nuclei into the controller, we recommend amplify 13 cycles for H3K27ac, and 12 cycles for H3K27me3. Cycle number should be adjusted based on sample type, histone modification, and the number of estimated recovered nuclei.

**Time taken**

- - Antibody + pA-Tn5 conjugation (A1-2): 1-1.5 hours
  - Nuclei preparation (A3): 1-1.5 hours
  - Epitope targeting (A4): overnight
  - Nuclei washing and tagmentation (B1-2): 2-2.5 hours
  - Droplet generation (B3): 30 mins
  - Reverse transcription and cell barcoding in droplet (B3): 2 hours
  - Products Cleanup (B4): 1 hour
  - Library pre-amplification (C): 1-2 hours
  - DNA / RNA library preparation (C): 3-4 hour
  - Library sequencing (D): time varies

**Anticipated results**

For final sequencing library, fragments in DNA libraries are typically between 200bp – 1,000bp with nucleosomes pattern, while fragments in cDNA libraries commonly show narrow peak pattern at 400-500bp (Fig. 1a-c).

**References**

1. Strahl, B. D. & Allis, C. D. The language of covalent histone modifications. Nature 403, 41–45 (2000).

2. Preissl, S., Gaulton, K. J. & Ren, B. Characterizing cis-regulatory elements using single-cell epigenomics. Nat. Rev. Genet. 24, 25–27 (2022).

3. Zeisel, A., Hochgerner, H., Lönnerberg, P., Johnsson, A., Memic, F., Van Der Zwan, J., ... & Linnarsson, S. (2018). Molecular architecture of the mouse nervous system. Cell, 174(4), 999-1014.
